# Aluminum as a Base for Artificial Teeth

**Published:** 1874-02

**Authors:** S. M. Furman

**Affiliations:** Leavenworth, Kansas.


### ARTICLE V.

### *Aluminium as a Base for Artificial Teeth.*

BY DR. S. M. FURMAN, OF LEAVENWORTH, KANSAS.

Read before the Kansas State Dental Association at Ottawa, Oct. 14, 1873.

Believing that this metal is strangely overlooked in the dental laboratory, the writer would endeavor to point out some of its advantages as demonstrated by his own experience, and meet some of the objections urged against it. The writer has used it only by Dr. Hollingsworth's method, and although it can be neatly manipulated in connection with rubber, a more perfect plate can be secured by his process than by swaging.

In enumerating the objections brought against cast plates we have—1st. The question of shrinkage. 2d. The danger of crushing the block and uncertainty of finishing the plate for the patient at the appointed time.

It is a well known fact that all materials used for bases, except swaged plates, shrink upon cooling, and rubber is no exception to the rule, as all who have seen a "rocking plate" will testify. With this metal, as with celluloid also, the blocks of teeth when closely fitted together admit of a vertical shrinkage *only*, and consequently the back teeth are drawn so much towards the center of the plate that the above mentioned abomination, a "rocking plate," is produced. This difficulty is obviated in Aluminium, as the spacing of each block before casting admits of a slight shrinkage in *all* directions without drawing or changing the form of the plate. I have never seen other than a perfect fit with this metal, with all the finest lines of the mouth faithfully represented.

To meet the difficulty of the crushing of the blocks in cooling, a delicate paste is introduced between the blocks before casting, which while being stable enough to prevent the flow of the molten metal into the joints, is compressed into so small a space as to be scarcely noticeable on removing from the flask. The paste is also used around the upper margins of the artificial gum, to prevent accident at the rim from the inevitable shrinkage.

That a perfect plate will always be secured, the writer cannot assert, but he has met with complete success in nearly every effort; when failures have come they were anticipated before opening the flask, and were the result of some departure from the directions of the inventor.

The durability and cleanliness of aluminium no one can deny; while its extreme lightness, being but one-seventh that of gold, and its freedom from discoloration, establish the value of this remarkable metal as a base.

That it will ever be used by itinerants as a cheap base at degrading prices, is impossible, as a skilled hand is necessary in carrying out the delicate manipulations in preparing such a denture for the mouth. Honorable men should give the public the benefit of this invention.

It takes less than one ounce of metal, ordinarily, to make a plate, while from ten to twelve hours is ample time for the whole process.—*Missouri Dental Journal.*

